# Retrospective study of cancer types in different ethnic groups and genders at Karachi

**DOI:** 10.1186/2193-1801-2-118

**Published:** 2013-03-19

**Authors:** Sheikh Abdul Khaliq, Syed Baqir Naqvi, Anab Fatima

**Affiliations:** Department of Pharmaceutics, Faculty of Pharmacy, Hamdard University, Karachi, Pakistan; Department of Pharmaceutics, Faculty of Pharmacy, University of Karachi, Karachi, Pakistan

**Keywords:** Ethnic groups, Cancer cases, Incidences of cancer

## Abstract

Retrospective study of Cancer types in different ethnic groups & genders determines the pattern of cancers in different ethnic groups & genders during the last eight years reported in Oncology wards of hospitals of Karachi, Pakistan. Every single one male & female case with histologically and cytologically established cancer was enrolled from January 2003 to December 2010. Data for all patients were collected retrospectively by patient’s file & charts, which represents the population of Karachi, Interior Sindh & Balochistan. 5134 patients (Male = 2432 / Female = 2702) investigated for their diagnosis of cancer type, ethnicity, age & gender. Classification of malignancy was done according to the International Classification of Disease coding system by W.H.O (ICD-10). The statistical analysis was performed for mean, standard error & proportions for ethnic groups & genders. Proportionately 47.37% males and among which major ethnic groups 17% Sindhi, 17% Immigrant, 4% Baloch, 3% Pukhtoon, ≈ 4% Punjabi, 1% Siraiki, 2% Minorities and 52.62% females, in which 16% Sindhi, 21% Immigrant, 4% Baloch 3% Pukhtoon, 5% Punjabi, 1% Siraiki, 3% Minorities. Mean age of males = 45.75 years, SE ± 0.227 and for females = 44.07, SE ± 0.183. The three most occurring tumors in all cancers of male were found Head & Neck, Adenoma/Carcinoma of Glands & Body cavity membranes, GIT, and females Breast, Head & Neck, Adenoma/Carcinoma of Glands & Body cavity membranes, GIT. The analysis of data indicates Head & Neck is most common cancer among male, in the similar way Breast cancer is the most common malignancy among female.

**Electronic supplementary material:**

The online version of this article (doi:10.1186/2193-1801-2-118) contains supplementary material, which is available to authorized users.

## Introduction

Adenomas, carcinomas or sarcomas are types of cancer which affect many people around the world. In Pakistan, being developing state, the cost of management of these cancers, not only affect individuals those suffer from it but affect their families and society at large. Financial burden is not only the issue but emotional disaster is greater than former. Pakistan is one of the countries of diversified types of ethnic groups which include Sindhis, Immigrants (migrated from India after 1947 and their descendents), Balochs, Pukhtoons, Punjabis, Kashmiris & others. Unfortunately, there is no single comprehensive data available to categorize the cancers and their types in these major ethnic groups. In countries where screening and reporting of cancer is excellent, they have made strategic alignment of their health resources to overcome the problem at earlier stage, which is not only reflected in their quality of life but also increased average life of individuals. The population of Pakistan is around 160.9 million, per capita income $2410 and life expectancy is 62 and 63 in male and female respectively while possibility of dyeing in 15–60 years is 218 and 194 in men and women respectively per 100,000 individuals. Pakistan has spent 2% of their GDP (Gross Development Product) on health sector ([Meropol and Schulman 2007]). Pakistan is unsuccessful historically to develop the database for cancer patients; the only source was developed by PMRC (Pakistan Medical & Research Council) as a single source (Bhurgri et al. [2002a], [b]). The KCR (Karachi Cancer Registry) was established in 1995 as one of the sample to gauge the burden of cancer in local population. KCR registered the cases of Karachi south from 1995 to 2005. Similarly APCR (Aga Khan University Pathological Based Cancer Registry) established in 2000, which covered initially 64 centers of Pakistan. The strategy was that to increase at least ten new centers per year to have massive effect on determining the actual incidences of cancer. The APCR and KCR combined approximation gives the frequency of 1998–2002 for Karachi population 9,802,134, among which 5.261,712 males and 4,540,422 females, yearly growth rate 3.52; Quetta population 759,245 (56% males and 44% females), yearly growth rate 4.13; Hyderabad population 2,840,653 (52.2% male, 47.8% female) yearly growth rate 1.13; Larkana, population 4,169,488 (51.8% male, 48.2% female) yearly growth rate 2.49 and Peshawar population 3,880,989 (52.2% male, 47.8% female) yearly growth rate 3.17. There is 30 to 40% incompleteness or variability of data but this is the nearest Pakistan which has been covered to evaluate ([Bhurgri et al. 2006], Bhurgri et al. [2002a], [b][, Jemal et al. 2005]). The present article provides a base to estimate the occurrence of cancers in different ethnic groups residing in Pakistan.

## Material and methods

This retrospective study conducted in Karachi, Pakistan, where material is collected from six different state of the art government and private hospitals located in Karachi and more than 5283 patients histopathologically diagnosed with any type of cancer and among which 5134 patients included in analysis for the period of 2003 to 2010. 149 patients were excluded because of preliminary diagnosis, lack of patient’s objective findings, diagnosis is not confirmed for any cancer, availability of incomplete data of patient like ethnic community, age, gender & belonging to geographic location and children ≤ 12 years. Majority of cases were received from state owned hospital at Karachi. The information is obtained from case sheets and patient’s file available in medical record room of the hospital. The data collected include age, gender, ethnic group, occupation, cancer type, date of diagnosis. SPSS software applied to collected data to calculate occurrence of cancer in each ethnic group and mean age gender wise and standard deviation (standard error).

## Observation and results

The findings of present study (Table 1) include Sindhis, Immigrants, Balochs, Pukhtoons, Punjabis, Siraikis & Other Minorities male & female from January 2003 to December 2010 suffering from any type of cancer, 5283 patients attended the oncology wards out of which 5134 (Male 2432 out of 2513/ Female 2702 out of 2770) were analysed & 149 (2.82%) were excluded from analysis due to incomplete information or loss of follow-up, 2432 (47.37%) being males, among which 864 (17%) Sindhi male, 850 (17%) Immigrant male, 223 (4%) Baloch male, 149 (3%) Pukhtoon male, 192 (≈ 4%) Punjabi male, 28 (1%) Siraiki male, 118 (2%) Minorities male and 2702 (52.62%) being females, in which 806 (16%) Sindhi female, 1062 (21%) Immigrant female, 230 (4%) Baloch female, 172 (3%) Pukhtoon female, 267 (5%) Punjabi female, 38 (1%) Siraiki female, 135 (3%) Minorities female. Mean age of all males were 45.75 years with SE ± 0.227 (Sindhi; 43.38, SE ± 0.473, Immigrants; 47.42, SE ± 0.459, Baloch; 46.80, SE ± 1.024, Pukhtoon; 45.50, SE ± 1.349, Punjabi; 49.51, SE ± 1.042, Siraiki; 39.43, SE ± 3.078, Minorities; 44.74, SE ± 1.490) and for females were 44.07 with SE ± 0.183 (Sindhi; 44.04, SE ± 0.467, Immigrant; 44.29, SE ± 0.374, Baloch; 42.59, SE ± 0.925, Pukhtoon; 43.12, SE ± 0.979, Punjabi; 45.28, SE ± 0.806, Siraiki; 44.45, SE ± 2.397, Minorities; 43.80, SE ± 1.067).

**Table 1:** **(Study population demographics)**

Ethnic group	Gender	No. of cases (%)	Mean age (Years)	Standar Error/Deviation
**Sindhi**	Male	864 (17%)	43.38	0.473
	Female	806 (16%)	44.04	0.467
**Immigrants**	Male	850 (17%)	47.42	0.459
	Female	1062 (21%)	44.29	0.374
**Baloch**	Male	223 (4%)	46.80	1.024
	Female	230 (4%)	42.59	0.925
**Pukhtoon**	Male	149 (3%)	45.50	1.349
	Female	172 (3%)	43.12	0.979
**Punjabi**	Male	192 (≈4%)	49.51	1.042
	Female	267 (5%)	45.28	0.806
Minorities*	Male	146 (3%)	44.74	1.490
	Female	173 (3%)	43.80	1.067
**Total cases**	Male	2432	45.75	0.227
	Female	2702	44.07	0.183

* Include Afghani, Parsi, Kashmiri, Catholic, Hindu & Unknown in the file.

**Figure 1:**
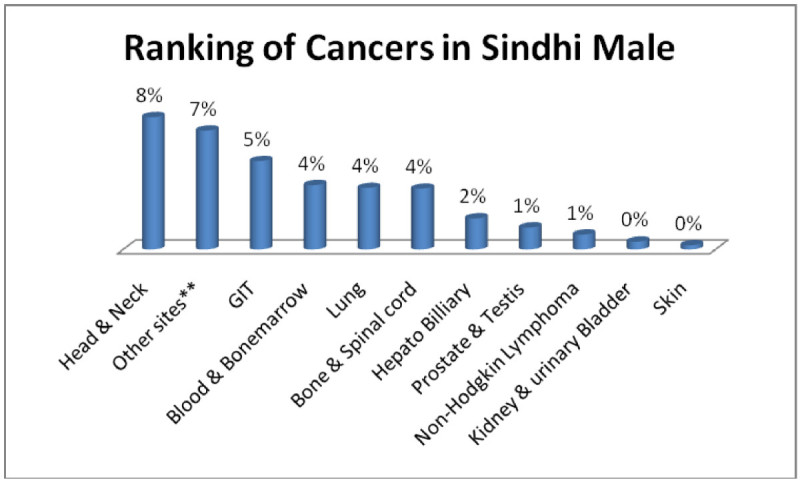
**Ranking of cancers in Sindhi male.**

**Figure 2:**
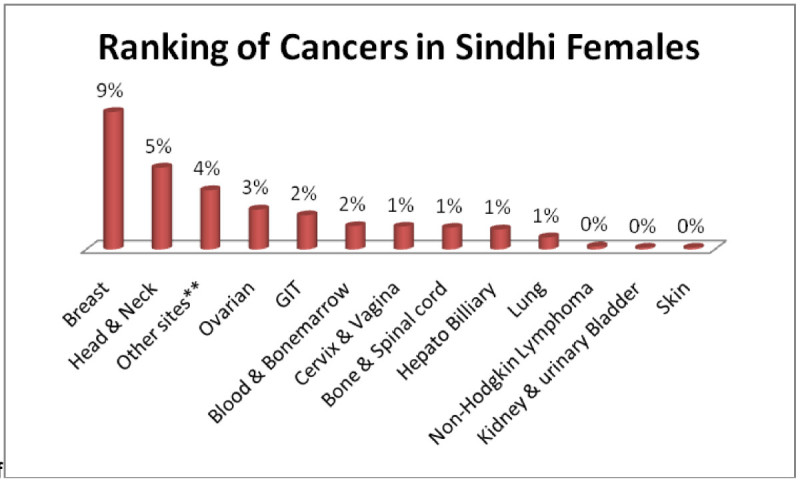
**Ranking of cancers in Sindhi females.**

The three most occurring tumors among in all cancers were found in Sindhi males; Head & Neck 8%, Adenoma/Carcinoma of Glands & Body cavity membranes 7%, GIT 5% (Figure 1) and among females three most common tumors, Sindhi females; Breast 9%, Head & Neck 5%, Adenoma/Carcinoma of Glands & Body cavity membranes 4% (Figure 2).

**Figure 3:**
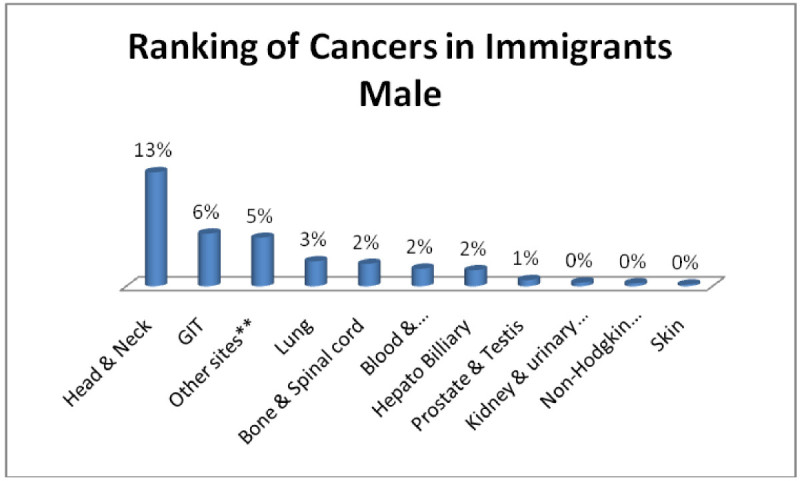
**Ranking of cancers in immigrants male.**

**Figure 4:**
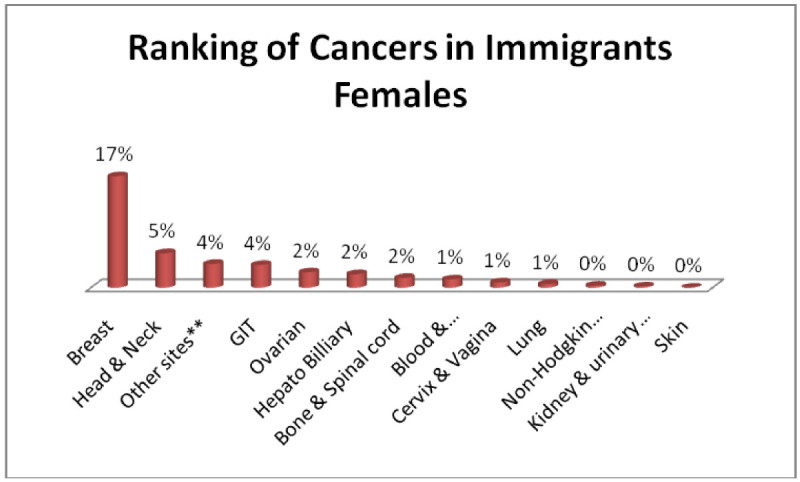
**Ranking of cancers in immigrants females.**

In the same way three most common cancers in immigrant males; Head & Neck 13%, GIT 6%, Adenoma/Carcinoma of Glands & Body cavity membranes 5% (Figure 3) and immigrant females; Breast 17%, Head & Neck 5%, Adenoma/Carcinoma of Glands & Body cavity membranes, GIT 4% each (Figure 4).

**Figure 5:**
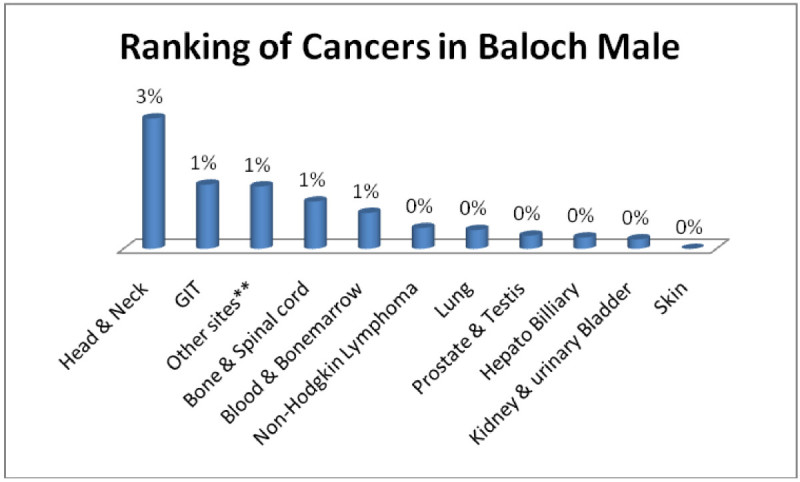
**Ranking of cancers in Baloch male.**

**Figure 6:**
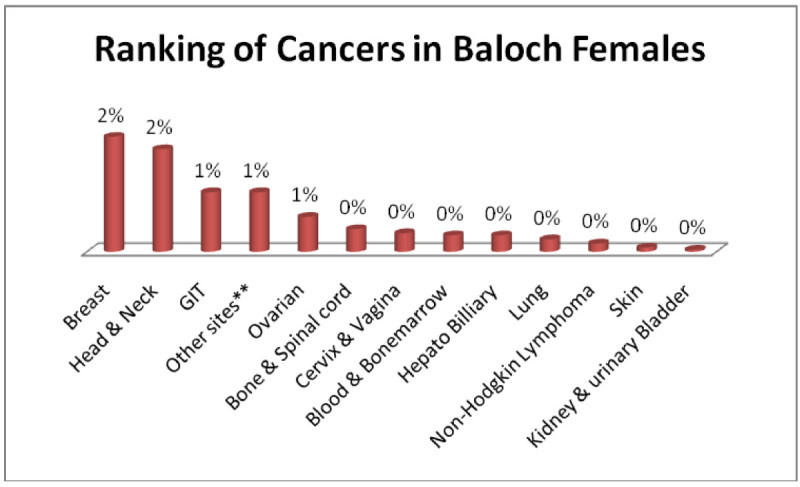
**Ranking of cancers in Baloch females.**

Similarly Baloch males; Head & Neck 3%, GIT, Adenoma/Carcinoma of Glands & Body cavity membranes, Bone & Spinalcord, Blood & Bonemarrow 1% each (Figure 5) and baloch females; Breast, Head & Neck 2% each, GIT, Ovarian, Adenoma/Carcinoma of Glands & Body cavity membranes 1% each (Figure 6).

**Figure 7:**
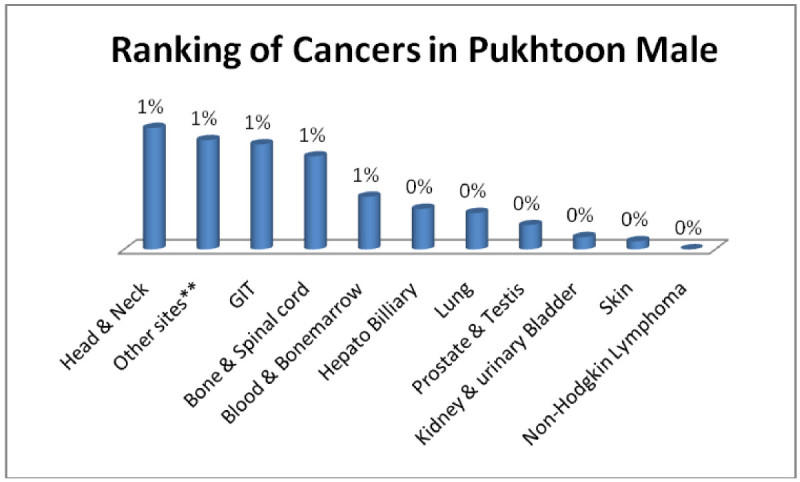
**Ranking of cancers in Pukhtoon male.**

**Figure 8:**
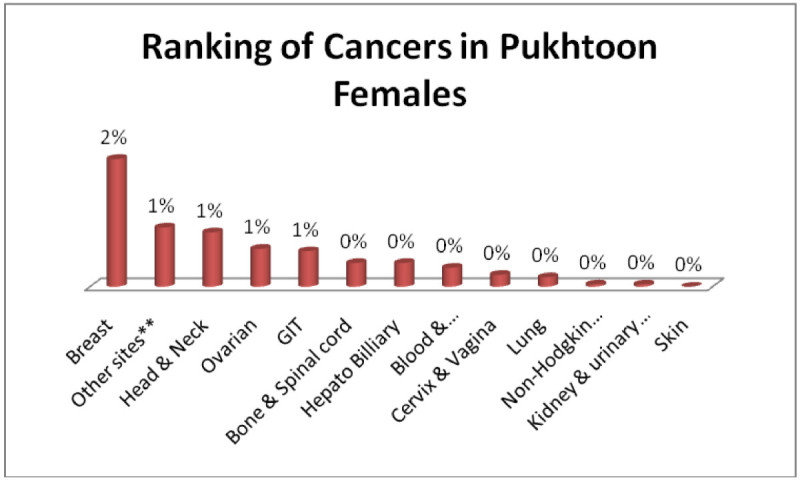
**Ranking of cancers in Pukhtoon females.**

Pukhtoon males; Head & Neck, GIT, Adenoma/Carcinoma of Glands & Body cavity membranes, Bone & Spinalcord, Blood & Bonemarrow 1% each (Figure 7) and pukhtoon females; Breast 2%, Adenoma/Carcinoma of Glands & Body cavity membranes, Head & Neck, Ovarian, GIT 1% each (Figure 8).

**Figure 9:**
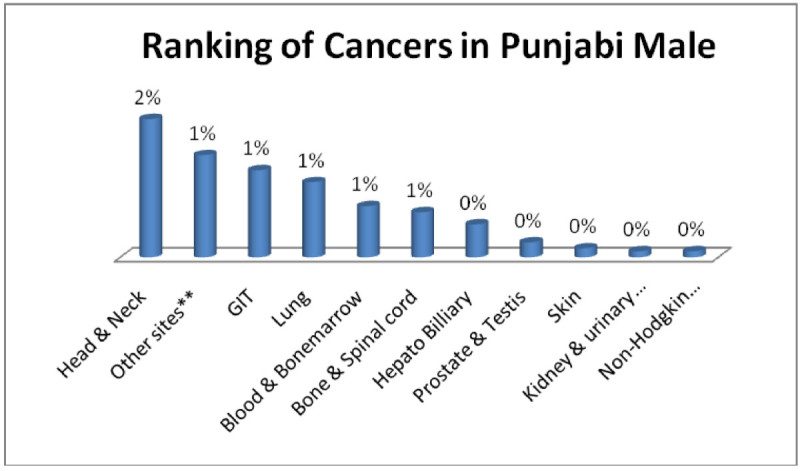
**Ranking of cancers in Punjabi male.**

**Figure 10:**
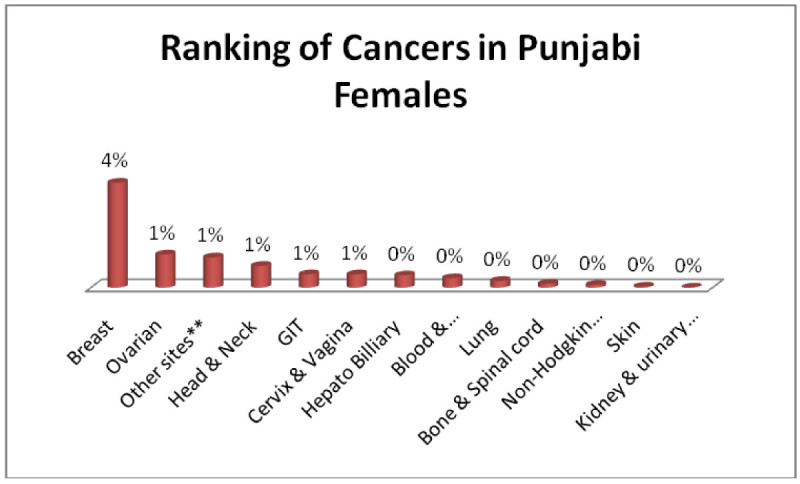
**Ranking of cancers in Punjabi females.**

Punjabi males; Head & Neck 2%, GIT, Adenoma/Carcinoma of Glands & Body cavity membranes, Bone & Spinalcord, Blood & Bonemarrow, Lung 1% each (Figure 9) and punjabi female; Breast 4%, Ovarian, Head & Neck, GIT, Adenoma/Carcinoma of Glands & Body cavity membranes, Cervix & Vagina 1% each, (Figure 10).

**Figure 11:**
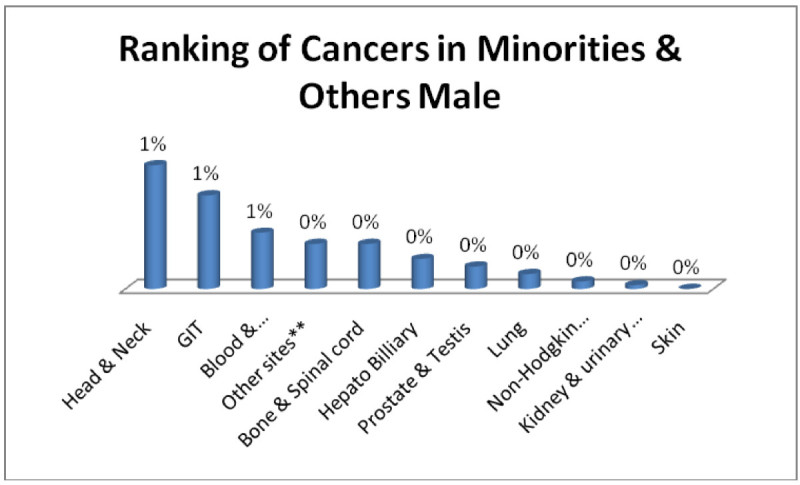
**Ranking of cancers in minorities & others male.**

**Figure 12:**
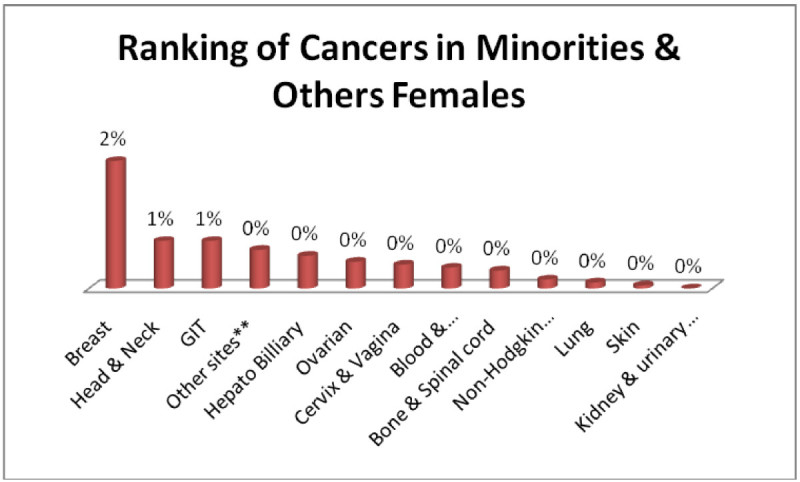
**Ranking of cancers in minorities & others females.**

And Minority males; Head & Neck, GIT, Blood & Bonemarrow 1% each (Figure 11) and minority females; Breast 2%, Head & Neck, GIT 1% each (Figure 12).

The analysis of data indicates Head & Neck is most common cancer among male ethnic groups of Pakistan, while Carcinoma of Glands & Body cavities membranes, GIT are the second most common cancers, in the similar way Breast cancer is the most common malignancy among all ethnic group’s female, while Head & Neck is second most common among females.

## Conclusion and discussion

The most alarming situation in Karachi is shift of mean age in last ten years of cancers of all sites from 51.2 years among male and 50.0 years among female ([Bhurgri et al. 2006]) to 45.75 years in males and 44.07 in female. This is clear indication of requirement of more resources for screening at earlier stage of cancers as well as awareness of early signs and symptoms among general population. Head and neck cancer is found to be the most prevalent cancer among all ethnic groups of Pakistan, which is ranked one in males and two in females. In head and neck cancers, oral cavity cancer in Karachi has the highest incidences reported worldwide, for 100,000 populations gender wise, the age standardized rate is 22.5 in males and 20.4 in females for the data of Karachi south from 1st January 1998 to 31st December 2002 ([Bhurgri et al. 2006]). The risk factors can be associated with this type of cancer includes the use of beatle nut, pan, tobacco chewing, use of naswar, pan masala, areca nut, gutka and smoking habits, however alcohol consumption is not prevalent in Pakistan, so should not be considered as risk factor unlike western countries. Most of the cases diagnosed and observed in patients file had history of usage of any one of said items or combination of them. One of the surveys in Karachi represents that 36% male and 44% females chew pan or pan with tobacco in Karachi ([Bhurgri et al. 2003][, Bhurgri 2005]).

In the similar way breast cancer is most prevalent among all ethnic groups and accounts one third of cancer in females and it has been observed that incidence of breast cancer in Karachi is highest in Asia except Israel ([Bhurgri et al. 2006]). In another study of 46 patients, where analysis has done on the base of age groups, the most common age group was 40–49 years with 14 cases (30.4%), then 50–59 years with 12 cases (26.0%), followed by 30–39 years with 10 cases (21.73%), 60–69 years 07 cases (15.21%), 20–29 years 1 case (2.17%), 70–79 years 1 case (2.17%) and there was also one case (2.17%) of 80 years and above age group ([Naeem et al. 2008]). Breast cancer is also found to be the most common disease in the middle age group 40–59 years ([Siddiqui et al. 2000][, Siddiqui and Rasool 2001][, Baloch and Iqbal 2006]), similar findings observed in the current study. Possible risk factors accounted for the development of breast cancer includes early menarche, late menopause, reproductive hormones, hormone replacement therapies, genetic factors (BRCA1 and BRCA2). Several studies conducted around the world include Malacia, Germany, USA and even India where breast cancer is detected at early stage because of better implementation of screening programs ([Kuraparthy et al. 2007][, Klonoff-Cohen et al. 1998][, Yip et al. 2006][, Arndt et al. 2001][, Oluwole et al. 2003]).

The rise in cases of GIT cancers especially colorectal cancer is really alarming, one of the retrospective analysis from 1995–1999 has noted 41% rise in the incidence in males ([Bhurgri et al. 2006]), however it does not mean that other cancers should be neglected, in Karachi, which is capital of Sindh province, many patients come from nearest vicinity which are ethnically sindhis and balochs, while majority of Karachi population comprises of immigrants, which demands more careful monitoring and screening of these said ethnic groups, as we can observe from the data that cancer of lung, liver, bone marrow and cervix require more focused probe to develop strategies to make possible to keep them at lower incidence rate.

Pakistan, being a developing country, scarce resources, need to develop strategies to reduce the burden of cancer in terms of financial and other losses. National Cancer Control Program (NCCP) should be implemented strictly and WHO, government and other health authorities including private sector NGOs should provide necessary resources to develop strategies for screening, prevention, diagnosis and treatment of cancers especially head and neck cancers, breast cancer, colorectal cancer and other prevailing cancers. Pharmaceutical companies should invest in cancer research to come up with new cost effective medications which are not only safe but having compelling evidences of efficacy. Some cancers can be minimized just by proper screening and immunization like in females above 18 years, if Pap test is used commonly as screening then we can prevent cancer of cervix at earlier stage, moreover now vaccine is also available as prophylaxis from cervix cancer. Similarly liver cancer progressed from hepatitis-B infection can be prevented by immunizing population by their vaccines. The possible strategy for prevention and early diagnosis of breast cancer is self and clinical examination of breast over age 20, mammography over age 40, similarly colorectal cancer fecal occult blood test over age 50 in male and flexible sigmoidoscopy, colonoscopy, double contrast barium enema over age 50 in females, for prostate cancer digital rectal exam and prostate specific antigen (PSA) over the age of 50. The government, NGOs and private sector should design and implement effective awareness campaigns for general public for the age specific symptoms and screening and to emphasize that early diagnosis can not only reduce economic burden but also families emotions and painful treatment.

## Authors’ original submitted files for images

Below are the links to the authors’ original submitted files for images. Authors’ original file for figure 1 Authors’ original file for figure 2 Authors’ original file for figure 3 Authors’ original file for figure 4 Authors’ original file for figure 5 Authors’ original file for figure 6 Authors’ original file for figure 7 Authors’ original file for figure 8 Authors’ original file for figure 9 Authors’ original file for figure 10 Authors’ original file for figure 11 Authors’ original file for figure 12
