# Supplementary figures and images for: Retrospective study of cancer types in different ethnic groups and genders at Karachi
Source: Springerplus. 2013 Mar 19;2(1):118. doi: 10.1186/2193-1801-2-118 (PMC3625419; doi:10.1186/2193-1801-2-118)

# Ranking of Cancers in Sindhi Male

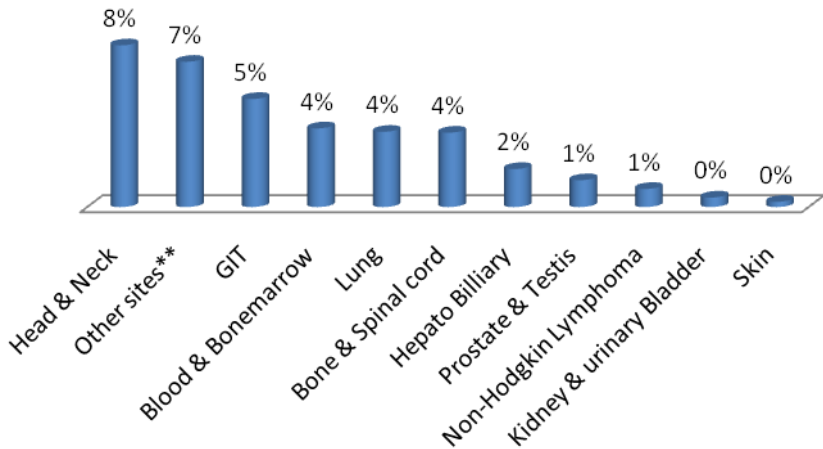

Supplement: Supplementary file 1 — Authors’ original file for figure 1 [file 40064_2012_201_MOESM1_ESM.pdf]

# Ranking of Cancers in Sindhi Females

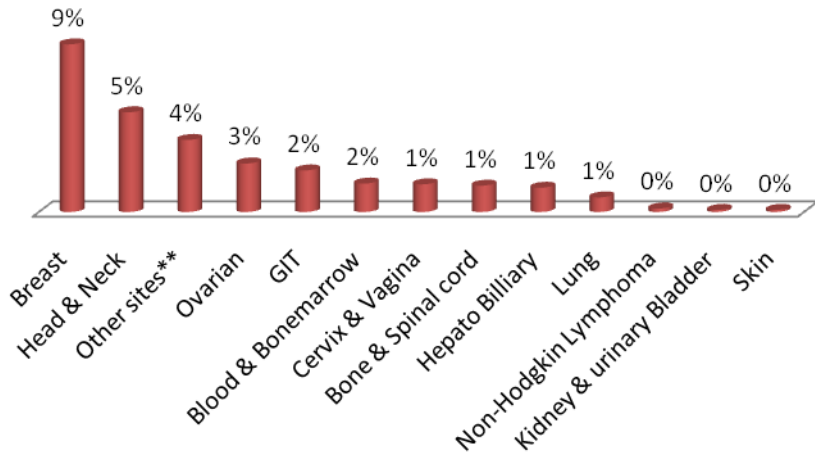

Supplement: Supplementary file 2 — Authors’ original file for figure 2 [file 40064_2012_201_MOESM2_ESM.pdf]

# Ranking of Cancers in Immigrants Male

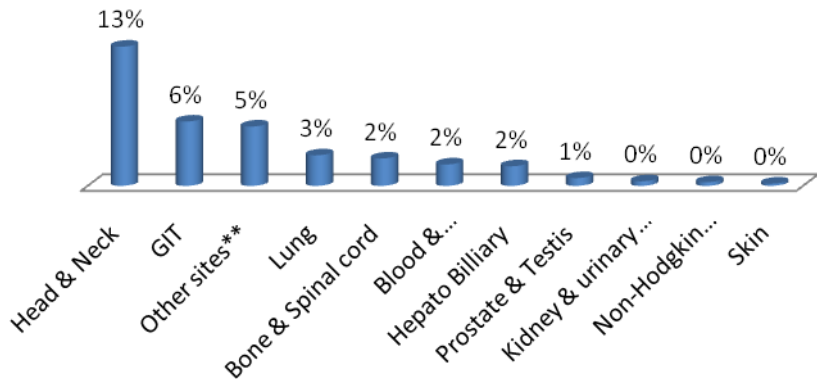

Supplement: Supplementary file 3 — Authors’ original file for figure 3 [file 40064_2012_201_MOESM3_ESM.pdf]

# Ranking of Cancers in Immigrants Females

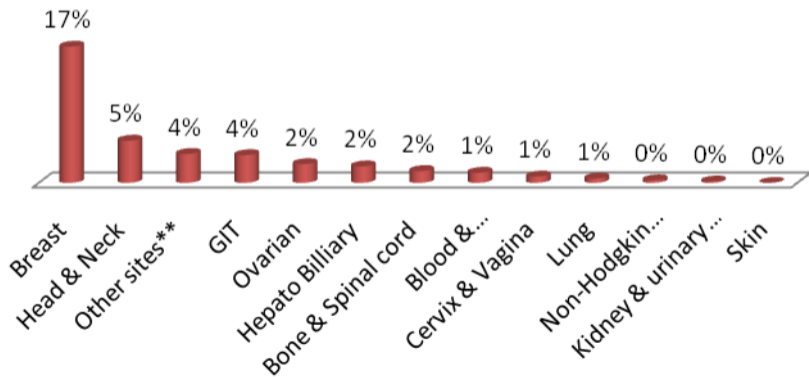

Supplement: Supplementary file 4 — Authors’ original file for figure 4 [file 40064_2012_201_MOESM4_ESM.pdf]

# Ranking of Cancers in Baloch Male

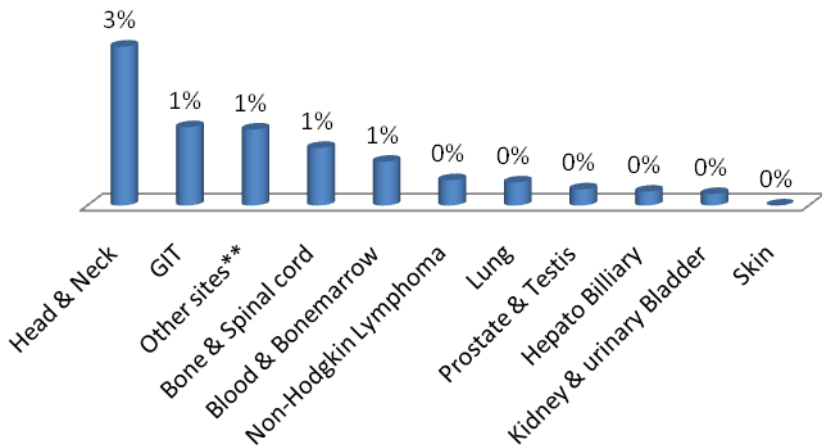

Supplement: Supplementary file 5 — Authors’ original file for figure 5 [file 40064_2012_201_MOESM5_ESM.pdf]

# Ranking of Cancers in Baloch Females

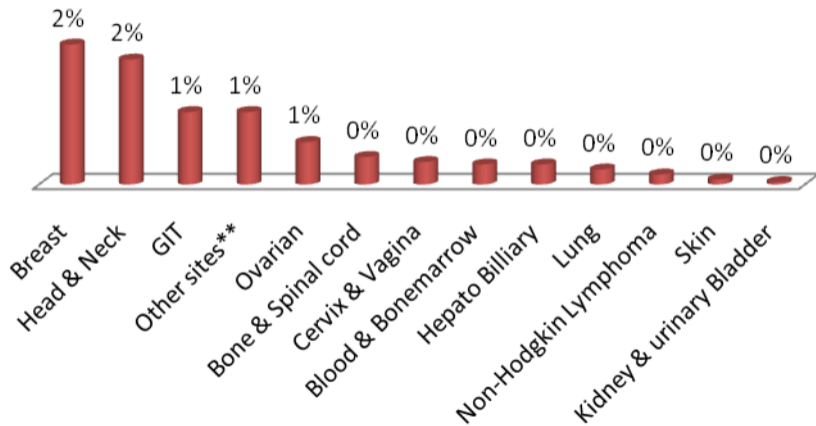

Supplement: Supplementary file 6 — Authors’ original file for figure 6 [file 40064_2012_201_MOESM6_ESM.pdf]

# Ranking of Cancers in Pukhtoon Male

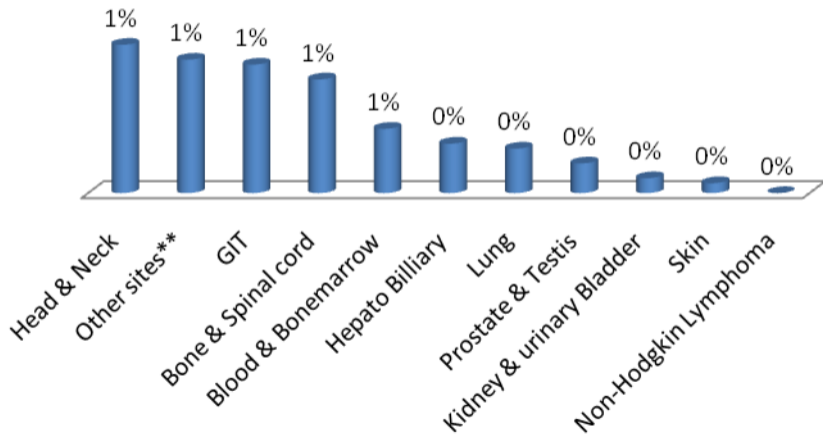

Supplement: Supplementary file 7 — Authors’ original file for figure 7 [file 40064_2012_201_MOESM7_ESM.pdf]

# Ranking of Cancers in Pukhtoon Females

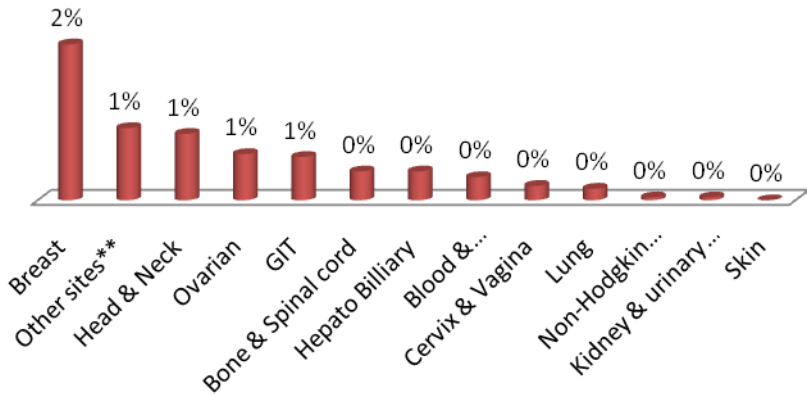

Supplement: Supplementary file 8 — Authors’ original file for figure 8 [file 40064_2012_201_MOESM8_ESM.pdf]

# Ranking of Cancers in Punjabi Male

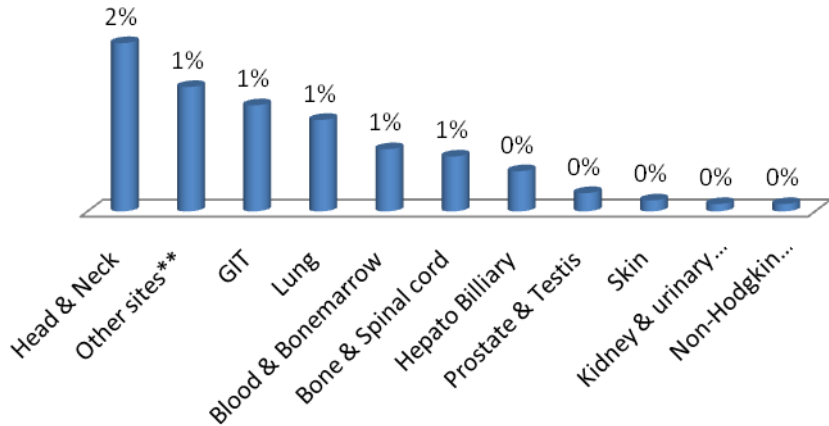

Supplement: Supplementary file 9 — Authors’ original file for figure 9 [file 40064_2012_201_MOESM9_ESM.pdf]

# Ranking of Cancers in Punjabi Females

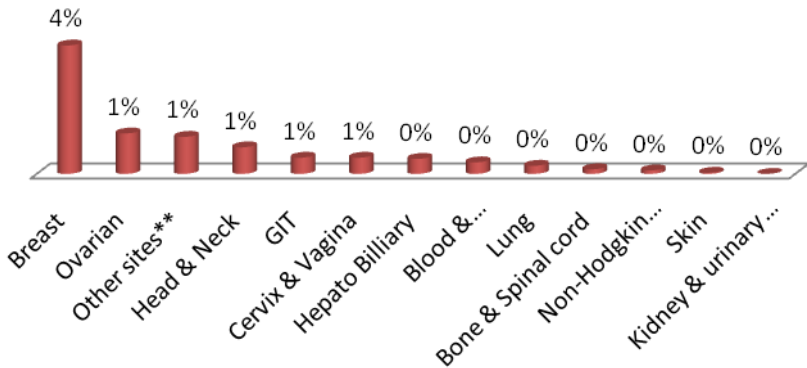

Supplement: Supplementary file 10 — Authors’ original file for figure 10 [file 40064_2012_201_MOESM10_ESM.pdf]

# Ranking of Cancers in Minorities & Others Male

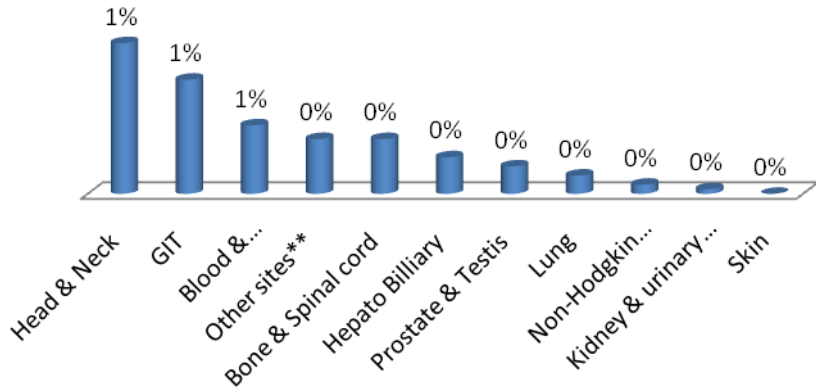

Supplement: Supplementary file 11 — Authors’ original file for figure 11 [file 40064_2012_201_MOESM11_ESM.pdf]

# Ranking of Cancers in Minorities & Others Females

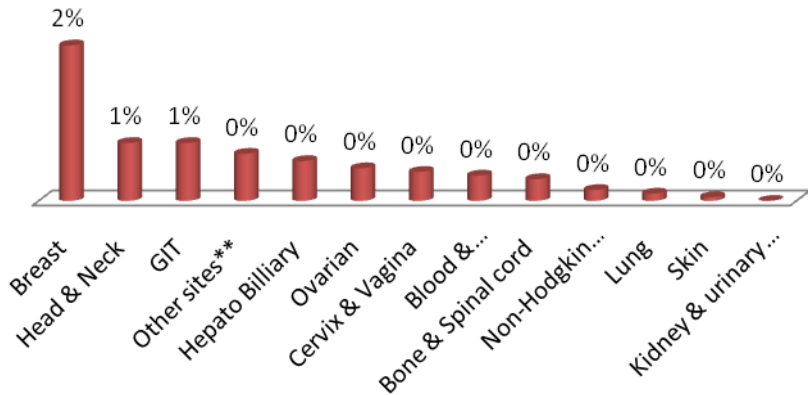

Supplement: Supplementary file 12 — Authors’ original file for figure 12 [file 40064_2012_201_MOESM12_ESM.pdf]
